# Identifying Critical Age Periods for the Prevention of Metabolic Complications in Obesity: An Integrative Analysis of Body Composition, Biochemical Profiles and Nutritional Recommendations in 29,544 Adults

**DOI:** 10.3390/nu18101533

**Published:** 2026-05-12

**Authors:** Irina A. Lapik, Inna Yu. Tarmaeva, Svetlana V. Klochkova, Dmitry B. Nikityuk

**Affiliations:** 1 Federal Research Centre of Nutrition, Biotechnology and Food Safety, 2/14 Ustinsky Proyezd, Moscow 109240, Russia; 2 Patrice Lumumba Peoples’ Friendship University of Russia, Moscow 117198, Russia; 3 I.M. Sechenov First Moscow State Medical University (Sechenov University), Ministry of Health of the Russian Federation, Moscow 119991, Russia

**Keywords:** obesity, body composition, bioelectrical impedance analysis, visceral fat, skeletal muscle, metabolic complications, age-specific prevention, dietary intake, chronic low-grade inflammation, oestradiol, menopausal transition, nutritional recommendations

## Abstract

**Background/Objectives:** Evidence-based nutritional recommendations for obesity management require understanding of sex-specific and age-specific body composition patterns and their associations with metabolic biomarkers, habitual dietary intake and chronic low-grade inflammation. This study aimed to characterize body composition phenotypes in a large clinical cohort of adults with obesity, to evaluate associated metabolic and inflammatory biomarker patterns, to contextualise these patterns against habitual nutrient intake assessed in a dietary subcohort, and to derive age- and sex-specific nutritional recommendations based on the identified patterns. **Methods:** We performed a cross-sectional analysis of 29,544 adults with obesity (BMI ≥ 30; 21,374 women, 8170 men; age 30–69) who underwent multi-frequency bioelectrical impedance analysis (BIA; InBody 770). Biochemical assessments (fasting glucose, lipid profile, uric acid, HbA1c, insulin) were available for 2019 hospitalized patients from the same population. Habitual dietary intake was quantitatively assessed in a dietary subcohort of 423 patients using the validated Russian software-based questionnaire “Scientific Nutrition Analysis Tool”. Inflammatory biomarkers (high-sensitivity CRP, IL-6) and adipokines (leptin, adiponectin) together with serum 25(OH)D were measured in an inflammation/adipokine subcohort of 116 patients. A body composition phenotype with low relative muscle mass and high visceral fat (VFA ≥ 100 cm^2^) was defined using FNIH criteria (ALM/BMI < 0.789 men, <0.512 women). Benjamini–Hochberg FDR correction (q < 0.05) was applied for multiple comparisons. **Results:** The body composition phenotype prevalence increased progressively with age: men 24.6% (30–39) to 42.0% (60–69); women 10.3% (30–39) to 31.8% (60–69). Skeletal muscle mass (SMM) was positively associated with uric acid (r = +0.347, *p* < 0.001, FDR q < 0.05) and inversely associated with HDL-cholesterol (r = −0.321, *p* < 0.001, FDR q < 0.05)—both associations with direct nutritional implications. BMI was associated with fasting insulin (r = +0.233, *p* < 0.001, FDR q < 0.05). Women showed significant age-related metabolic differences between the 30–39 and 60–69 age groups: fasting glucose +12.9%, triglycerides +34.8%, uric acid +15.0% (all *p* < 0.001); in men, significant differences were observed for fasting glucose (+7.0%) and HbA1c (+5.2%) (both *p* < 0.001), while lipid parameters did not reach significance. In the dietary subcohort, habitual saturated-fat intake exceeded recommended values in 70–72% of patients of both sexes, dietary fibre intake was below recommended levels in 73–85%, and habitual calcium intake decreased significantly with age in women (1022 → 746 mg/day, *p* = 0.028). Serum CRP was elevated (median 5.59 mg/L, *n* = 59). In a separate extended laboratory subcohort, serum oestradiol declined markedly with age in women (55.0 → 16.8 pmol/L between 30–39 and 50–59 years, *p* < 0.001), consistent with the menopausal transition; serum testosterone in men remained stable across age groups; and 25(OH)D insufficiency (<30 ng/mL) was prevalent in 49.7–55.8% of patients. **Conclusions:** The identified sex-specific and age-specific body composition patterns provide a rationale, supported by observed dietary and inflammatory patterns, for targeted nutritional intervention: increased dietary protein, omega-3 fatty acids supplementation, low-glycemic-index dietary patterns, and purine restriction with hyperuricemia. Routine BIA-based nutritional screening combined with quantitative dietary assessment should begin at age 30, with preventive monitoring at age 40 and intensification of control at age 50, to guide personalized dietary planning in obesity.

## 1. Introduction

Obesity has reached pandemic proportions globally, affecting over 2.1 billion adults as of 2021, with projections suggesting this number will exceed 3.8 billion by 2050 [1]. In the Russian Federation, the multi-centre population-based ESSE-RF study reported the prevalence of obesity (BMI ≥ 30 kg/m^2^) at approximately 26.6% in men and 30.8% in women aged 25–64 years, with a clear age-dependent rise across decades and considerable regional variation [2]. The corresponding public-health burden—including cardiovascular disease, type 2 diabetes mellitus and non-alcoholic fatty liver disease—provides the immediate clinical context for the present study. The clinical significance of obesity is determined not merely by excess fat accumulation but by its heterogeneous metabolic consequences, including dyslipidemia, insulin resistance, chronic low-grade inflammation, and cardiovascular disease [3,4]. Several physiological mechanisms underlie the metabolic deterioration observed in middle-aged and older adults with obesity. First, age-related sarcopaenia—characterised by reduced muscle protein synthesis, anabolic resistance to amino acid stimulation and infiltration of intramuscular adipose tissue—leads to a measurable decline in skeletal muscle mass and function from approximately the fifth decade. Second, hormonal changes, particularly the decline of oestrogens during the menopausal transition in women and a progressive decline in testosterone in men, drive a redistribution of adipose tissue from gluteo-femoral to visceral and ectopic depots. Third, the post-menopausal milieu accelerates loss of bone mineral density and shifts the balance of inflammatory cytokines, contributing to so-called “meta-inflammation”—a chronic, low-grade, immunometabolic inflammatory state that has recently been characterised at the systems level as both a driver of metabolic dysfunction and, at early stages, a partly adaptive response [5]. Adipokine and adipo-protease signalling (e.g., chemerin, neprilysin, adipolin) provides further evidence that the SMI–VFA axis reflects systemic metabolic and inflammatory regulation rather than purely structural body composition changes [6]. Conventional anthropometric assessment using body mass index (BMI) has been increasingly recognized as insufficient for capturing the complexity of obesity-related metabolic risk, as it fails to differentiate fat from lean mass or to assess adipose tissue distribution [7]. The 2025 Lancet Diabetes & Endocrinology Commission on obesity classification proposed a paradigm shift, integrating body fat distribution and metabolic biomarkers into the diagnostic framework [8]. Consensus criteria for the phenotype characterized by the coexistence of excess adiposity and reduced skeletal muscle mass—often termed sarcopenic obesity in the literature—have been established, requiring both body composition and functional muscle assessment [9,10]. The ESPEN/EASO expert group highlighted that structured obesity management, including incretin-based pharmacotherapy, may lead to variable muscle mass loss [11,12]. The novelty of the present study, in comparison with the existing international literature, lies in three aspects: the use of one of the largest single-centre BIA-based clinical cohorts of adults with obesity reported to date (n = 29,544), drawn from a specialised national tertiary nutrition centre; the integration of BIA-derived body composition data with biochemical biomarkers, with quantitative habitual dietary intake assessed using a standardised Russian software-based protocol; and with directly measured inflammatory biomarkers (CRP, IL-6) and adipokines (leptin, adiponectin) and explicit sex- and age-stratified description in the Russian adult population. The aim of this study was to perform an integrative analysis of body composition parameters, metabolic and inflammatory biomarkers, and habitual dietary intake in a large clinical population of adults with obesity to perform the following: characterize the relationships between BIA-derived body composition parameters and the body composition phenotype (low relative muscle mass combined with high visceral fat), including sex-specific and age-related patterns; analyse correlations between body composition and metabolic biomarkers and identify age periods showing the most pronounced cross-sectional differences in metabolic risk markers; and contextualise these against habitual nutrient intake patterns to derive sex- and age-targeted nutritional recommendations.

## 2. Materials and Methods

### 2.1. Study Design and Population

This multi-cohort observational study utilized data from the Federal Research Centre for Nutrition, Biotechnology and Food Safety (Moscow, Russia), collected between 2021 and 2026. This is a retrospective cross-sectional analysis. With 29,544 participants, the study had >80% power to detect correlations of r ≥ 0.05 at α = 0.05. Complete-case analysis was performed for the biochemical subcohort. Patients were referred to the Centre—a tertiary national obesity-management referral centre—from primary-care outpatient clinics, endocrinologists and dieticians across the Russian Federation; recruitment therefore reflects a clinical referral cohort rather than a population-based or registry-based sample. The study comprised four cohorts:

Cohort 1 (BIA Cohort, *n* = 29,544): A retrospective analysis of body composition assessments in patients with obesity (BMI ≥ 30 kg/m^2^), aged 30–69 years, performed using the InBody 770 multi-frequency bioimpedance analyser (InBody 770, Biospace, Seoul, Republic of Korea). The cohort included 21,374 women (72.3%) and 8170 men (27.7%). Mean age was 53.8 ± 10.2 years.

Cohort 2 (Biochemistry Cohort, *n* = 2019): Biochemical assessments were performed only for patients who were hospitalized (inpatient care). BIA was available for both inpatient and outpatient settings. The biochemistry subcohort (*n* = 2019) therefore represents a consecutive sample of hospitalized patients from the same population. For correlation analyses, only the first biochemical assessment per patient was used to avoid bias from repeated measurements.

Cohort 3 (Dietary subcohort, *n* = 423): habitual dietary intake was quantitatively assessed in a subsample of 423 patients (323 women, 100 men; mean age 42.8 ± 12.7 years; mean BMI 40.3 ± 8.0 kg/m^2^) at admission, using the standardised, validated Russian software-based questionnaire “Scientific Nutrition Analysis Tool” (Scientific Nutrition Analysis Tool, Federal Research Centre of Nutrition, Biotechnology and Food Safety, Moscow, Russia), developed and used at the Federal Research Centre for Nutrition, Biotechnology and Food Safety. The programme combines 24 h-recall and food-frequency components, automatically calculates mean daily intake of energy, macronutrients, fatty-acid classes (saturated, mono- and polyunsaturated, *n*-3, *n*-6, *n*-9, trans-fatty acids), free sugars, dietary fibre, cholesterol, purines, fructose, individual amino acids (including leucine, lysine, methionine, tryptophan), vitamins and minerals, and quantifies food-group consumption. All dietary data refer to habitual home intake prior to hospitalisation.

Cohort 4 (Inflammation and adipokine subcohort, *n* = 116): a subset of women (*n* = 105) and a small group of men (*n* = 11) aged 20–69 years with obesity in whom serum high-sensitivity CRP, interleukin-6 (IL-6), leptin, adiponectin, fasting insulin, total IgE and serum 25(OH)–vitamin D were measured by standard immunochemical methods together with the routine biochemistry panel.

Inclusion criteria: age 30–69 years, diagnosed obesity (BMI ≥ 30 kg/m^2^), signed informed consent. Exclusion criteria: type 1 diabetes mellitus, pregnancy, severe hepatic or renal disease, thyroid pathology, hormonal therapy. The study was approved by the Ethics Committee of the Federal Research Centre of Nutrition, Biotechnology and Food Safety (Protocol No. 2021-05/17, approved 15 March 2021).

Socio-demographic and lifestyle characteristics of the BIA cohort. All patients were residents of the Russian Federation, predominantly from the Central Federal District (Moscow and the Moscow region). The educational profile was characterised by a predominance of higher (university-level) education and a smaller proportion with secondary education only; self-reported household income was predominantly middle-tier (sufficient for basic needs but not for major purchases). Occupationally, the cohort was homogeneous and consisted of patients of occupational category I according to the Russian physiological labour classification (predominantly intellectual/desk-based work)—corresponding to a physical activity coefficient of 1.4 × basal metabolic rate, i.e., very low habitual physical activity. No patients reported engagement in heavy physical labour or in regular structured exercise programmes at the time of assessment. The occupational homogeneity of the cohort, while limiting external generalisability to physically active populations, simultaneously reduces residual confounding by occupational physical activity in the present age- and sex-stratified analyses.

Integrated cohort with full data overlap. A subset of patients had complete patient-level data for the principal modalities used in this study—BIA, biochemistry, habitual dietary intake, inflammatory biomarkers (high-sensitivity CRP, IL-6), adipokines (leptin, adiponectin), and the hormonal panel (oestradiol or testosterone, 25(OH)-vitamin D). Within this integrated subset, patient-level cross-modality correlations between dietary, inflammatory and body-composition parameters were analysed. Where the four principal subcohorts (BIA, biochemistry, dietary, and inflammation/adipokine) overlap only partially at the individual-patient level, this is acknowledged explicitly throughout the Results, and the relevant analyses are flagged as cohort-level rather than patient-level.

### 2.2. Body Composition Assessment

Body composition was assessed using the InBody 770 multi-frequency bioimpedance analyser (Biospace, Seoul, Republic of Korea), employing a tetrapolar 8-point tactile electrode system with measurements at six frequencies (1, 5, 50, 250, 500, and 1000 kHz) across five body segments. All measurements were performed in the morning, after a 12 h fast, following 10 min of supine rest, with exclusion of physical activity for 24 h prior to assessment, in accordance with standardized BIA protocols [13,14]. Three diagnostic approaches for identifying low relative muscle mass were compared in sensitivity analysis: SMI-based: SMI (SMM/weight × 100) < 29.0% for men and <22.9% for women; ALM/BMI-based (FNIH criteria): appendicular lean mass/BMI < 0.789 for men and <0.512 for women; Population-adjusted: SMI below −1 SD of the sex-specific mean. Functional muscle assessment (handgrip strength, chair-stand test, and gait speed) was not available; the body composition phenotype as defined here therefore represents a screening category and cannot be equated with clinically defined sarcopenic obesity per ESPEN/EASO criteria.

### 2.3. Body Composition Phenotype Definition

We defined the body composition phenotype of interest as the simultaneous presence of: obesity (BMI ≥ 30 kg/m^2^); low relative muscle mass by FNIH criteria (ALM/BMI < 0.789 in men, <0.512 in women) and high visceral adiposity (VFA ≥ 100 cm^2^). Functional muscle assessment was not available. Our analysis is therefore limited to body composition parameters.

### 2.4. Biochemical Assessment

In Cohort 2, venous blood samples were collected after a 12 h fast. Parameters were analysed on the Cobas c 702 automated analyser (Roche Diagnostics, Mannheim, Germany): fasting glucose (mmol/L; CV < 2.0%), glycated haemoglobin (HbA1c, %; CV < 1.5%), total cholesterol (mmol/L; CV < 2.0%), LDL-cholesterol (mmol/L; CV < 2.5%), HDL-cholesterol (mmol/L; CV < 2.5%), triglycerides (mmol/L; CV < 2.0%), alanine aminotransferase (ALT, U/L; CV < 3.0%), aspartate aminotransferase (AST, U/L; CV < 3.0%), uric acid (µmol/L; CV < 2.5%), creatinine (µmol/L; CV < 2.0%), and urea (mmol/L; CV < 2.5%). Fasting insulin (µIU/mL; CV < 5.0%) was measured by electrochemiluminescence immunoassay (Elecsys Insulin, Roche Diagnostics, Mannheim, Germany). HOMA-IR was calculated as fasting glucose × fasting insulin/22.5. In Cohort 4, high-sensitivity C-reactive protein (hs-CRP), interleukin-6 (IL-6), leptin, and adiponectin were measured by enzyme-linked immunosorbent assay (ELISA) using commercial kits (R&D Systems, Minneapolis, MN, USA). Total IgE was measured by chemiluminescent immunoassay (Siemens Healthineers, Erlangen, Germany). Serum 25(OH)-vitamin D was measured by ELISA using a 25-Hydroxy Vitamin D EIA kit (Immunodiagnostic Systems, Boldon, UK). Serum insulin and C-peptide were measured by ELISA using Insulin ELISA and C-Peptide ELISA kits (DRG Instruments GmbH, Marburg, Germany).

### 2.5. Dietary Assessment

In Cohort 3, habitual dietary intake was assessed at admission by trained nutrition specialists using the standardised, computer-based Russian questionnaire-software “Scientific Nutrition Analysis Tool” (Scientific Nutrition Analysis Tool, Federal Research Centre of Nutrition, Biotechnology and Food Safety, Moscow, Russia). The programme integrates 24 h-recall and food-frequency components and automatically computes mean daily intake of energy, macro- and micronutrients, fatty-acid classes, free sugars, dietary fibre, cholesterol, purines, fructose, individual amino acids, vitamins, and minerals, and food-group consumption. Reference daily intake values used for comparison were taken from the Russian methodological recommendations on physiological energy and nutrient requirements for adults (MP 2.3.1.0253-21 [15]). “Deficiency” and “excess” categories were defined as habitual intake below 80% or above 120% of the recommended value, respectively, applied separately for men and women in line with sex-specific norms. Habitual physical activity was characterised by occupational category (uniformly category I, physical activity coefficient 1.4 × BMR; see Section 2.1); structured leisure-time exercise, smoking and detailed alcohol consumption beyond the questionnaire item were not systematically collected.

### 2.6. Statistical Analysis

Statistical analysis was performed using IBM SPSS Statistics version 27.0 (IBM Corp., Armonk, NY, USA), R version 4.3.1 (R Foundation for Statistical Computing, Vienna, Austria), and Python 3.12 with SciPy 1.13 (Python Software Foundation, Wilmington, DE, USA). Normality of distribution was assessed using the Shapiro–Wilk test. Data are presented as median and interquartile range (Me [Q1; Q3]) or mean ± SD. The Mann–Whitney U test was used for two-group comparisons; the Kruskal–Wallis test with Bonferroni correction was used for multiple group comparisons; Spearman’s rank correlation coefficient (r_s) was used for correlation analysis. Statistical significance was set at *p* < 0.05. Benjamini–Hochberg False Discovery Rate (FDR) correction was applied using the p.adjust function (method = “BH”) in base R; all correlations reported as significant had FDR q < 0.05. Given the very large sample size of Cohort 1, statistical significance does not necessarily imply clinical relevance; effect sizes (correlation coefficients) are therefore reported and discussed alongside *p*-values, and weak associations (|r| < 0.20) are flagged accordingly throughout the Results.

## 3. Results

### 3.1. General Characteristics of the BIA Cohort

The general characteristics of the BIA cohort (*n* = 29,544) are presented in Table 1. The study population demonstrated a mean BMI of 40.4 ± 7.9 kg/m^2^, with mean VFA of 229.6 ± 48.7 cm^2^—substantially exceeding the critical threshold of 100 cm^2^, indicating pronounced visceral adiposity. Mean PBF was 46.9 ± 6.9%, approximately 1.5–2 times the upper physiological limit. The ECW/TBW ratio (0.384 ± 0.010) exceeded the threshold of 0.380, suggesting subclinical fluid overload—a feature consistent with chronic low-grade inflammation in obesity, although direct inflammatory biomarkers in this cohort were available only in the inflammation/adipokine subcohort (Cohort 4) and are reported separately in Section 3.6.

### 3.2. Correlation Analysis of Body Composition Parameters

**Figure 1:**
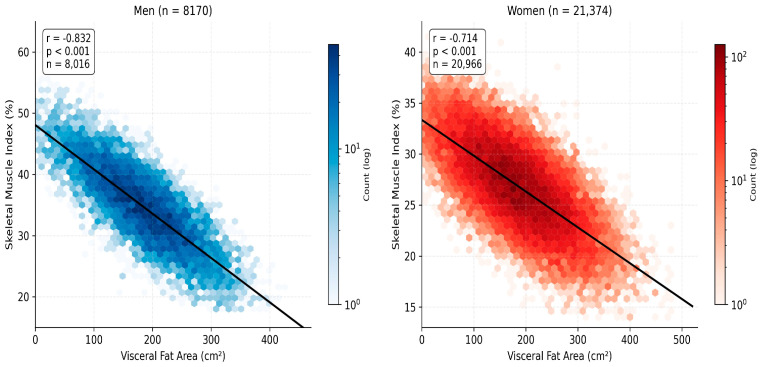
Spearman correlation matrix of body composition parameters in patients with obesity (*n* = 29,544). Colour intensity reflects correlation strength. All shown correlations had *p* < 0.001 and FDR q < 0.05.

**Figure 2:**
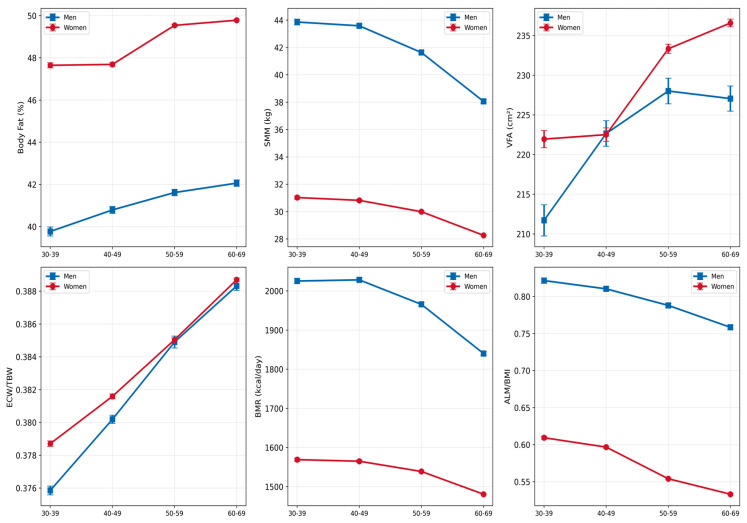
Scatter plots of SMI versus VFA by sex with linear regression lines. Each dot represents a patient (random subsample of 2000 per panel for visualization). Spearman r values and 95% CI are shown for men (blue) and women (red). Both associations: *p* < 0.001, FDR q < 0.05.

Spearman correlation analysis revealed strong inverse associations between SMI and VFA in both sexes: men (r = −0.837, *p* < 0.001, FDR q < 0.05) and women (r = −0.713, *p* < 0.001, FDR q < 0.05; Figure 1). In men, SMI also showed strong inverse correlations with ECW/TBW (r = −0.532, *p* < 0.001, FDR q < 0.05) and BMI (r = −0.827, *p* < 0.001, FDR q < 0.05), reflecting the coupling of relative muscle depletion with visceral adiposity and fluid overload. In women, SMI demonstrated an inverse correlation with BMI (r = −0.801, *p* < 0.001, FDR q < 0.05) and a positive association with FFM (r = +0.261, *p* < 0.001, FDR q < 0.05). VFA was positively correlated with ECW/TBW in both sexes (men: r = +0.394; women: r = +0.259; both *p* < 0.001, FDR q < 0.05), suggesting that visceral fat accumulation is accompanied by subclinical inflammatory fluid redistribution. These patterns are illustrated in Figure 2 and are consistent with the SMI–VFA axis as the central structural feature of obesity-related body composition dysregulation.

### 3.3. Body Composition Parameters by Obesity Class

**Figure 3:**
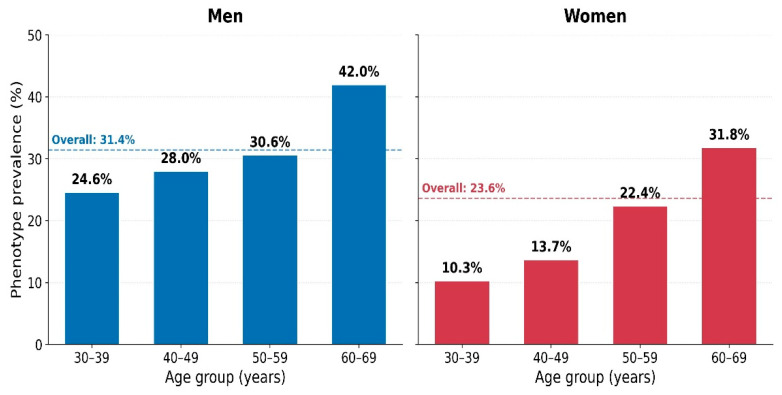
Prevalence of the body composition phenotype (low relative muscle mass + VFA ≥ 100 cm^2^) by sex and age group using FNIH criteria (ALM/BMI). Percentage labels shown above bars.

The body composition phenotype (low relative muscle mass + VFA ≥ 100 cm^2^) showed clear sex-specific and age-related patterns (Figure 3). The overall phenotype prevalence was 31.4% in men and 23.6% in women. In men, prevalence increased progressively with age: 24.6% (30–39), 28.0% (40–49), 30.6% (50–59), and 42.0% (60–69 years). In women, a similar but delayed pattern was observed: 10.3% (30–39), 13.7% (40–49), 22.4% (50–59), and 31.8% (60–69 years); the steepest increase was observed between the 40–49 and 50–59 decades.

### 3.4. Associations Between Body Composition and Metabolic Biomarkers

In women, between the 30–39 and 60–69 age groups, fasting glucose differed by +12.9%, triglycerides by +34.8%, uric acid by +15.0%, and HbA1c by +7.5% (all *p* < 0.001, Kruskal–Wallis). In men, fasting glucose (+7.0%) and HbA1c (+5.2%) showed significant age-related differences (*p* < 0.001), while lipid parameters did not reach significance. Integrated correlation analysis of paired BIA and biochemical data (*n* = 2019) revealed that SMM was positively associated with serum uric acid (r = +0.347, *p* < 0.001, FDR q < 0.05) and inversely associated with HDL-cholesterol (r = −0.321, *p* < 0.001, FDR q < 0.05); BMI was positively associated with fasting insulin (r = +0.233, *p* < 0.001, FDR q < 0.05). Given the very large sample size, additional correlations of weak magnitude (|r| ≤ 0.20) reached statistical significance but are unlikely to be of clinical importance; these are reported in Table 2 with explicit effect-size labels.

**Figure 4:**
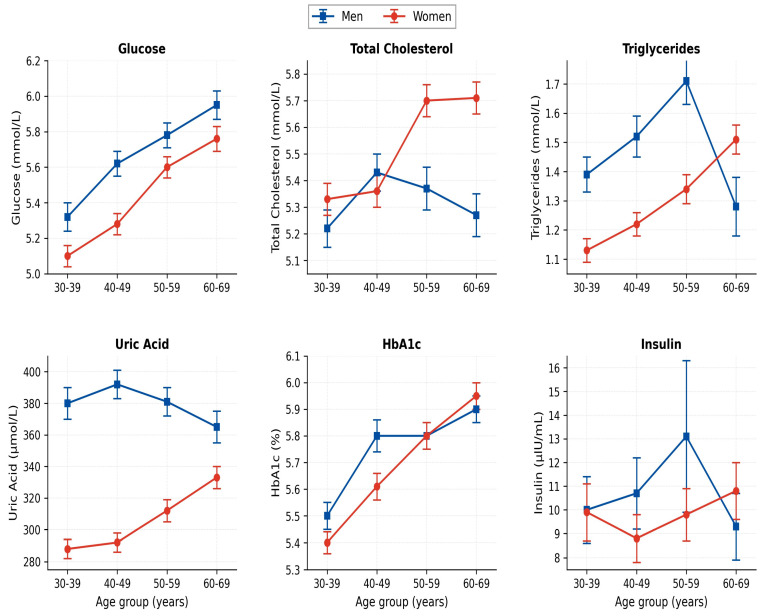
Age-related differences in metabolic biomarkers in patients with obesity by sex.

Median values of fasting glucose, total cholesterol, triglycerides, uric acid, HbA1c, and fasting insulin by age group and sex in patients with obesity (biochemistry cohort, *n* = 2019). Data are shown in Figure 4.

### 3.5. Habitual Dietary Intake in the Dietary Subcohort (n = 423)

**Figure 5:**
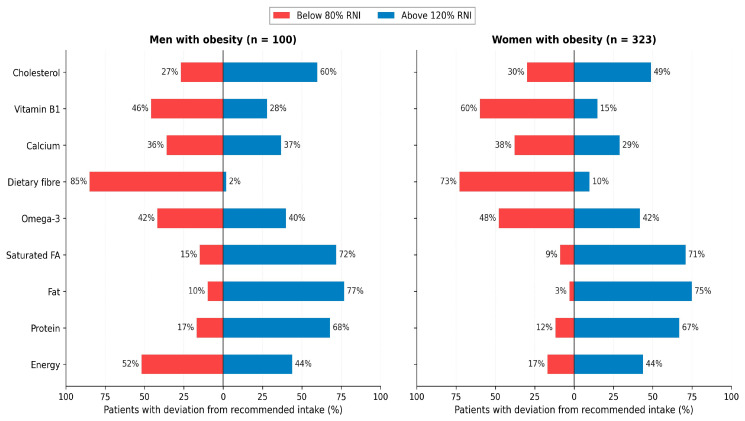
Habitual nutrient intake compared with Russian recommended values (MP 2.3.1.0253-21). For each nutrient, horizontal bars show the proportion of patients with habitual intake below 80% (deficiency, left, red) or above 120% (excess, right, blue) of the sex-specific reference value, separately for men (left panel, *n* = 100) and women (right panel, *n* = 323). Habitual saturated-fat excess and dietary-fibre deficit dominate in both sexes; calcium, magnesium, vitamin B1 and iron deficits are more prevalent in women.

Habitual dietary intake was assessed in 423 patients with obesity (323 women, 100 men; mean age 42.8 ± 12.7 years; mean BMI 40.3 ± 8.0 kg/m^2^). Sex-stratified intake values are summarised in Table 3 and the proportion of patients with intake outside the Russian recommended range is shown in Table 4 and Figure 5. Habitual energy intake (median 2700.8 kcal/day in men vs. 2192.1 kcal/day in women, *p* = 0.001) substantially exceeded the recommended energy intake for sedentary-to-moderately-active adults in both sexes. Median daily intakes of total fat (men 141 g; women 109 g) and saturated fatty acids (men 47.4 g; women 34.3 g) were well above recommended values, with saturated fat intake exceeding norms in 75.6% of men and 72.1% of women. Conversely, dietary fibre intake was insufficient in the majority of patients (85.0% of men below 25 g/day; 72.7% of women below 20 g/day; median 11.5 g/day in men and 10.6 g/day in women). Vitamin B1, calcium, magnesium and niacin were below recommended values in approximately one-third to one-half of patients, with greater rates of deficiency observed in women.

Sex-stratified comparison showed that men with obesity habitually consumed significantly higher absolute amounts of energy, protein, total and saturated fat, omega-3 fatty acids, calcium, magnesium, iron and B-vitamins than women (all *p* < 0.05; Table 3). When expressed as proportions exceeding or falling below recommended intake (Table 4 and Figure 5), the qualitative pattern is similar in both sexes—habitual intake is characterised by simultaneous excess of saturated fat, total fat and dietary cholesterol, with insufficient dietary fibre, vitamin B1 and, in women, calcium, magnesium and iron.

**Figure 6:**
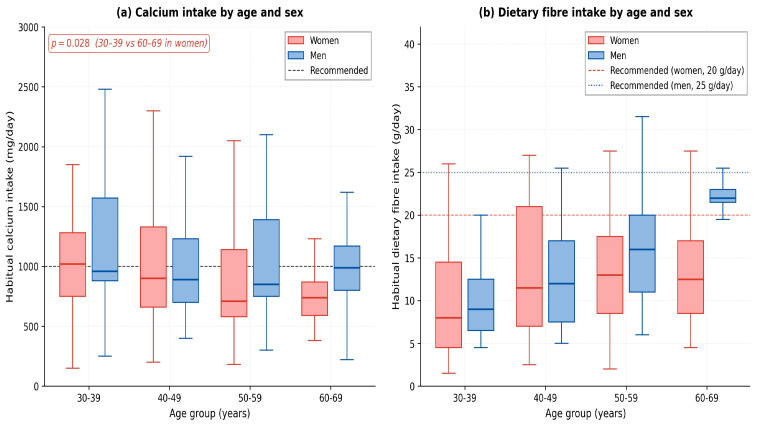
Age-related differences in habitual nutrient intake. (**a**) Habitual calcium intake by age group and sex; horizontal dashed line shows the recommended intake (1000–1200 mg/day). In women, calcium intake declines significantly between the 30–39 and 60–69 decades (*p* = 0.046). (**b**) Habitual dietary fibre intake by age group and sex; reference lines show recommended intake of 25 g/day (men) and 20 g/day (women). Both sexes show insufficient intake in the younger groups, with progressive improvement with age.

Habitual dietary intake also varied with age, with the most pronounced differences observed in women (Figure 6). In women, energy intake decreased from a median of 2815 kcal/day in the 30–39 group to 2037 kcal/day in the 60–69 group (Δ = −27.6%, *p* = 0.017), fat intake from 132 to 94 g/day (Δ = −28.7%, *p* = 0.003) and calcium intake from 1012 to 710 mg/day (Δ = −29.8%, *p* = 0.046), while dietary fibre intake increased from 8.0 to 15.3 g/day (Δ = +91.6%, *p* = 0.004). In men, age-related differences in habitual intake did not reach statistical significance (Kruskal–Wallis *p* > 0.10 for all macronutrients), although the smaller male subsample reduces statistical power.

In the subset of patients with extended dietary data, additional intake parameters of direct relevance to obesity-related metabolic risk were quantified. Median dietary purine intake was 567 mg/day in men and 412 mg/day in women, with the upper quartile reaching 748 mg/day in men—values consistent with the upper end of recommended limits and relevant to the SMM–uric acid association observed in the biochemistry cohort. Median dietary vitamin D intake was 3.2 μg/day in men and 2.3 μg/day in women, both well below recommended values (10–15 μg/day). Median leucine intake was 7.4 g/day in men and 6.4 g/day in women (*p* = 0.016). The mean ratio of plant-derived to total food mass was 50.1% in men and 58.8% in women, with a clear age-related rise in the proportion of plant-derived foods in both sexes.

### 3.6. Inflammatory Biomarkers, Adipokines and Serum Vitamin D (n = 116)

**Figure 7:**
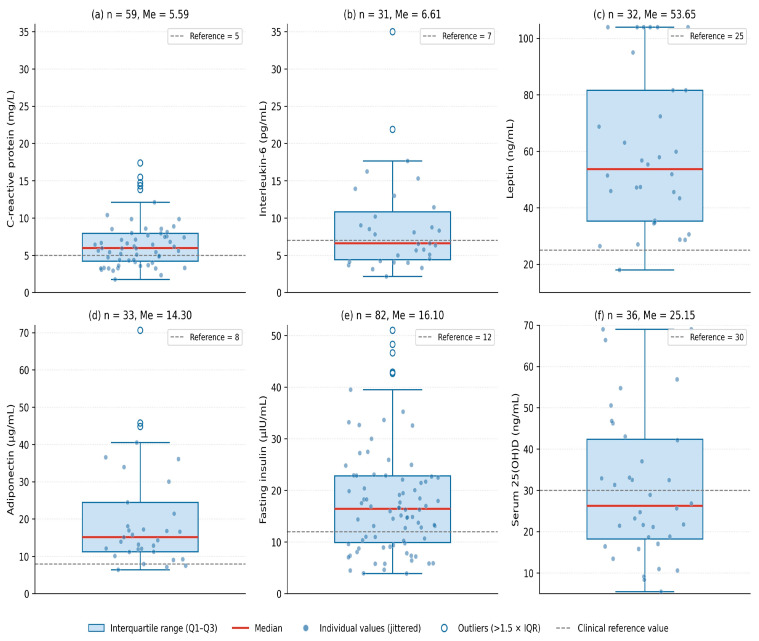
Inflammatory biomarkers, adipokines and serum 25(OH)D in adults with obesity (Cohort 4, *n* = 116). Box-plots show median and interquartile range with individual values overlaid; dashed reference lines indicate clinically relevant cut-offs. (**a**) High-sensitivity CRP, with most values exceeding the low-cardiovascular-risk threshold of 3 mg/L. (**b**) IL-6, with the median above the upper limit of normal. (**c**) Leptin, markedly elevated relative to lean reference values, consistent with obesity-related leptin resistance. (**d**) Adiponectin, distributed within the lower part of the reference range. (**e**) Fasting insulin, with values consistent with hyperinsulinaemia. (**f**) Serum 25(OH) D, with most values below the sufficiency threshold of 30 ng/mL.

In the inflammation/adipokine subcohort (Cohort 4, *n* = 116; 105 women, 11 men; predominantly women, results presented for the female subset where appropriate), serum high-sensitivity CRP, IL-6, leptin, adiponectin, fasting insulin, total IgE and serum 25(OH)D were measured at admission. Distributions are summarised in Figure 7 and Table 5.

Median high-sensitivity CRP was 5.59 mg/L (*n* = 59), exceeding the low-cardiovascular-risk threshold of 3 mg/L in the majority of patients and consistent with chronic low-grade inflammation. Median IL-6 was 6.6 pg/mL (*n* = 31), at the upper end of the reference range. Leptin (median 53.7 ng/mL) was markedly elevated relative to typical lean reference values, while adiponectin was distributed within the lower part of the reference range (median 14.6 μg/mL). Fasting insulin (median 16.1 μIU/mL) was consistent with hyperinsulinaemia. Serum 25(OH)D was below the sufficiency threshold of 30 ng/mL in the majority of patients (median 25.1 ng/mL, n = 31).

Spearman correlation analysis revealed clinically meaningful associations between inflammatory biomarkers and metabolic parameters. Serum CRP was positively correlated with serum uric acid (r = +0.30, *p* = 0.024), triglycerides (r = +0.34, *p* = 0.023) and BMI (r = +0.28, *p* = 0.058, borderline) and inversely with HDL-cholesterol (r = −0.35, *p* = 0.020). Fasting insulin was inversely correlated with HDL-cholesterol (r = −0.44, *p* < 0.001) and positively correlated with fasting glucose (r = +0.38, *p* = 0.002), serum uric acid (r = +0.43, *p* < 0.001) and triglycerides (r = +0.29, *p* = 0.022). Leptin was positively correlated with BMI (r = +0.41, *p* = 0.019) and fasting glucose (r = +0.41, *p* = 0.021). These directly measured associations support the inflammatory and adipokine-mediated component of obesity-related metabolic dysregulation suggested by the indirect ECW/TBW findings in Cohort 1 and complement the indirect markers used in earlier sections.

### 3.7. Hormonal Status and Micronutrient Profile by Age and Sex (n = 2019)

To complement the findings on inflammatory biomarkers and habitual dietary intake, the hormonal and vitamin–mineral status of patients with obesity was characterised in an extended laboratory subcohort (*n* = 2019 patients aged 30–59 years), stratified by sex and age decade. Sex- and age-specific values of insulin, TSH, parathyroid hormone, prolactin, oestradiol and testosterone are summarised in Table 6. The vitamin and mineral profile (25(OH)D, vitamin B12, folate, serum iron, potassium, sodium, chloride, calcium and magnesium) is summarised in Table 7.

The most striking hormonal finding was the marked age-related decline of serum oestradiol in women, from a median of 55.0 pmol/L in the 30–39 age group to 16.8 pmol/L in the 50–59 age group (Δ = −69.5%, *p* < 0.001), consistent with the menopausal transition. A parallel decrease in serum prolactin was observed in women (279.0 → 198.0 mIU/L, *p* < 0.001). Serum testosterone in men remained stable across age groups (9.71 → 9.96 nmol/L; *p* = 0.536). Fasting insulin and TSH did not show significant age-related changes within either sex, consistent with the persistence of compensatory hyperinsulinaemia throughout the adult age range studied. Serum parathyroid hormone showed a borderline-significant increase with age in women (*p* = 0.038), in keeping with the post-menopausal shift in calcium–bone homeostasis.

Insufficiency of serum 25(OH)–vitamin D (<30 ng/mL) was prevalent across the cohort, affecting 55.8% of men and 49.7% of women, with overt deficiency (<20 ng/mL) in 20.4% of all subjects. Median 25(OH)D values in men declined modestly from 29.0 ng/mL in the 30–39 group to 27.4 ng/mL in the 50–59 group; in women, median values were close to the lower bound of sufficiency throughout. Serum vitamin B12, folate and iron remained largely within reference ranges, with median ferritin in women showing a notable rise after the menopausal transition (31.2 → 50.6 ng/mL, *p* < 0.001), consistent with cessation of menstrual losses. Serum calcium showed a small but statistically significant fluctuation across age groups in women (*p* < 0.001), while no significant changes were observed for serum magnesium in either sex.

## 4. Discussion

The present analysis of 29,544 adults with obesity demonstrates that BIA-derived body composition profiling, when integrated with metabolic biomarker data, reveals sex-specific and age-specific patterns of cardiometabolic risk that cannot be captured by BMI alone.

### 4.1. The Muscle–Fat Axis and Metabolic Crosstalk

The strong inverse associations between SMI and VFA (men: r = −0.832; women: r = −0.714; both *p* < 0.001, FDR q < 0.05) quantify the reciprocal relationship between relative skeletal muscle depletion and visceral fat accumulation in obesity. This is consistent with the pathophysiological framework in which adipose tissue-derived inflammatory mediators (TNF-α, IL-6, resistin) are associated with muscle catabolism through NF-κB signaling, while reduced muscle mass is associated with lower metabolic rate [16,17,18]. In the present study, the inflammatory link is supported not only by indirect proxies (the elevated ECW/TBW ratio in the BIA cohort) but also by direct measurement in Cohort 4: median high-sensitivity CRP of 5.59 mg/L exceeded the low-CV-risk threshold of 3 mg/L in the majority of patients, and CRP was significantly correlated with serum uric acid (r = +0.30), triglycerides (r = +0.34) and HDL-C (r = −0.35), consistent with the metaflammation framework recently characterised at the systems level [5] and with the adipokine/adipo-protease evidence relating chemerin, neprilysin and adipolin to anthropometric and metabolic parameters in obesity [6]. The stronger correlation in men may reflect the higher visceral fat deposition tendency in men (android fat distribution).

The sex difference in body composition phenotype prevalence (31.4% in men vs. 23.6% in women) is consistent with known differences in body composition between sexes. In women, the delayed onset of phenotype prevalence increase (sharp rise after 50 years) is consistent with the described protective role of oestrogen on muscle mass and accelerated body composition changes associated with menopause [9,11], we underline that menopausal status was inferred from age and not measured.

The positive association between SMM and serum uric acid (r = +0.347, *p* < 0.001, FDR q < 0.05) is clinically relevant. Hyperuricemia is an independent cardiovascular risk factor, and the present data indicate it is associated with visceral fat distribution rather than overall obesity severity. Serum uric acid is, however, also strongly influenced by dietary factors—purine intake, fructose, alcohol, and protein source. Habitual dietary purine intake in our dietary subcohort was distributed around the upper recommended limit, with a non-trivial fraction of men exceeding it; because the dietary subcohort and the biochemistry subcohort are not paired patient-by-patient, the relative contributions of dietary purines and visceral adiposity to serum uric acid cannot be disentangled here. We therefore explicitly do not attribute the SMM–uric-acid association exclusively to body composition and acknowledge habitual diet as a likely co-determinant. The inverse association between SMM and HDL-cholesterol (r = −0.321, *p* < 0.001, FDR q < 0.05) is consistent with the role of skeletal muscle in reverse cholesterol transport through upregulation of ABCA1 and lipoprotein lipase activity [19].

### 4.2. Age Periods Showing Most Pronounced Cross-Sectional Differences

**Figure 8:**
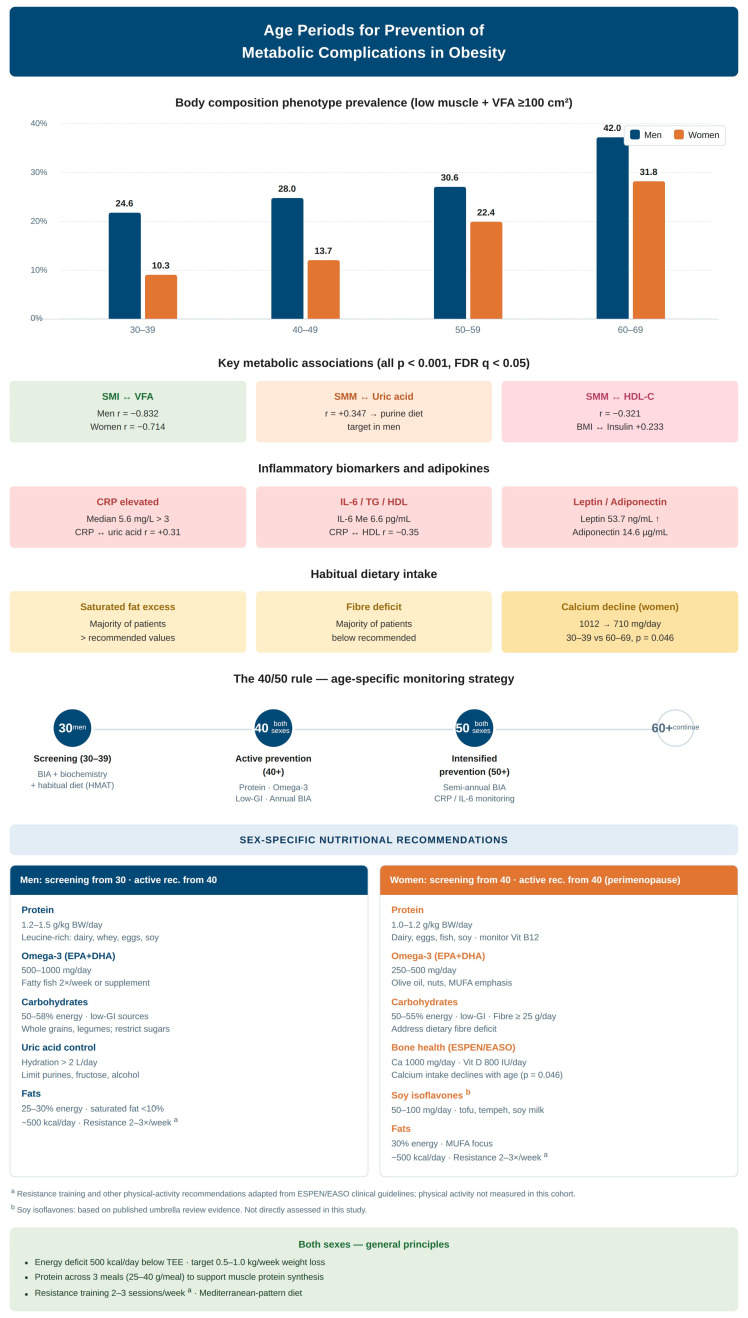
Conceptual, hypothesis-generating three-stage age-specific prevention framework for metabolic complications in obesity. This figure does not present empirical results of the present study. It is a conceptual schematic that integrates the cross-sectional findings of this work with established ESPEN/EASO clinical recommendations [9,20] to motivate prospective evaluation; the depicted age thresholds and stage-specific measures are proposed for hypothesis generation and should not be interpreted as validated clinical decision rules. The framework integrates BIA, biochemistry, habitual dietary intake and inflammatory biomarkers. Coloured panels denote Stage 1 (screening, from age 30 in men and from age 40 in women), Stage 2 (active prevention from age 40 in both sexes) and Stage 3 (intensified prevention from age 50 in both sexes). Vertical dotted lines link each stage to the corresponding age threshold on the timeline. The lower panel summarises the principal cross-sectional findings of the present study that support this framework. Resistance-training and other physical-activity recommendations are adapted from ESPEN/EASO clinical guidelines [9,20].

Integration of body composition, biochemistry, habitual dietary intake, inflammatory biomarkers and the hormonal/micronutrient profile identifies age periods in which differences in metabolic risk markers are most pronounced, differentiated by sex (Figure 8). The framework summarised in Figure 8 is presented as a conceptual, hypothesis-generating synthesis: it integrates the cross-sectional patterns observed in this cohort with established ESPEN/EASO recommendations and is intended to motivate prospective evaluation rather than to define validated clinical thresholds. We deliberately frame these as “age periods showing most pronounced cross-sectional differences” rather than as “critical windows” for prevention, since the cross-sectional design precludes causal inference: the present data are hypothesis-generating with respect to optimal timing of preventive intervention and require prospective confirmation. In men with obesity, the body composition phenotype prevalence increases progressively from 24.6% at 30–39 to 42.0% at 60–69 years, supporting the hypothesis that screening should begin no later than age 40, with the 50–59 decade representing a candidate period for intensified metabolic monitoring. In women, the most pronounced cross-sectional differences were observed in the 50–59 decade, with phenotype prevalence rising to 22.4% (+8.7 percentage points from the 40–49 decade), and concomitant differences in glucose, triglycerides and HbA1c—a pattern biologically anchored by the directly measured 69.5% decline of serum oestradiol between the 30–39 and 50–59 age groups (55.0→16.8 pmol/L, *p* < 0.001) (Section 3.7), consistent with the menopausal transition. The simultaneous observation, in women, of significantly declining habitual calcium intake (1022→746 mg/day, *p* = 0.028), elevated CRP across age groups, and a borderline rise of parathyroid hormone underlines the rationale for proactive nutritional and metabolic management in this period.

### 4.3. Phenotype-Specific Nutritional Recommendations Informed by Observed Dietary and Inflammatory Patterns

The dietary subcohort and the inflammation/adipokine subcohort jointly provide a quantitative description of habitual nutrient intake and chronic low-grade inflammation against which the body composition and biomarker findings can be contextualised. Several patterns are directly relevant to the recommendations developed below. Saturated-fat intake exceeded recommended values in 70–72% of patients of both sexes, motivating an emphasis on substitution toward unsaturated fatty-acid sources. Habitual dietary fibre intake was below recommended levels in approximately three-quarters of patients of both sexes. A non-trivial fraction of patients (~30%) had habitual omega-3 intakes below 80% of the recommended value, supporting recommendations for increased dietary intake or supplementation, particularly in patients with dyslipidaemia or elevated CRP. Low-glycemic-index dietary patterns are also recommended for glycaemic control and metabolic risk reduction [21]. In women, habitual calcium intake decreased significantly with age and dietary vitamin D intake was insufficient (median 2.3 μg/day)—a pattern of direct relevance to bone health in the post-menopausal period and providing observational support for explicit calcium and vitamin D recommendations. Habitual purine intake reached the upper end of recommended limits in a substantial proportion of men, providing observational support for purine moderation in the context of hyperuricaemia management. Elevated CRP and IL-6 in Cohort 4 provide a direct mechanistic anchor for anti-inflammatory dietary patterns (Mediterranean-style diets, omega-3-rich foods, fibre-rich plant-based components). For perimenopausal and postmenopausal women, soy isoflavone interventions may also support bone metabolism [22].

Building on the identified sex-specific and age-related body composition patterns, we propose nutritional recommendations aligned with the ESPEN practical guidelines for clinical nutrition in obesity and published expert consensus [9,20], and additionally supported by the observed dietary deficiencies and inflammatory pattern in the present cohort. These recommendations are intended to support dietary counselling and conventional food choices. The proposed phenotype-specific nutritional recommendations are summarised in Table 8. Based on the identified age-related patterns, a three-stage prevention framework is proposed and summarised in Table 9.

### 4.4. Limitations

Several limitations should be acknowledged. First, the cross-sectional design precludes causal inference regarding age-related changes in body composition and metabolic biomarkers; all associations are descriptive and cross-sectional. Second, the single-centre referral design may limit generalizability (the cohort is enriched in higher-BMI categories—mean BMI 40.4—compared with the general obese population) to other populations. Third, functional muscle assessment (handgrip strength, chair stand test, and gait speed) was not performed; therefore, our body composition phenotype represents a screening category based on body composition parameters only, and cannot be equated with clinically defined sarcopenic obesity as per ESPEN-EASO criteria [23]. Fourth, although high-sensitivity CRP, IL-6, leptin, adiponectin and serum 25(OH)D were measured directly in Cohort 4, the inflammation/adipokine subcohort was small, predominantly female, and not paired patient-by-patient with the BIA or dietary subcohorts, which limits stratified mechanistic analysis; classical inflammatory biomarkers were not measured in Cohorts 1 or 2. Fifth, the biochemistry subcohort (*n* = 2019) was limited to hospitalized patients, which may limit generalizability to the broader outpatient population with obesity. Sixth, dietary intake was assessed in a separate subcohort (*n* = 423) and was not paired patient-by-patient with biochemistry or with the inflammation subcohort, so within-patient diet–biomarker correlations could not be tested; the dietary subcohort was constructed by harmonising four internal databases of habitual intake with possible inter-database variability in completeness, and physiologically implausible values were screened and treated as missing. Seventh, although serum oestradiol and testosterone were measured directly in the extended laboratory subcohort and provide quantitative biological support for the menopausal-transition framework, individual menopausal status (years since last menstrual period, FSH–LH profile) was not formally diagnosed, and the residual confounding by individual hormonal trajectories is acknowledged. Eighth, structured leisure-time physical activity, smoking and detailed alcohol consumption were not systematically captured; the cohort was, however, occupationally homogeneous (predominantly intellectual/desk-based work, occupational category I, physical activity coefficient 1.4), which limits residual confounding by occupational physical activity. Ninth, additional contextual socio-demographic information beyond educational level, self-reported income and federal-district residence (e.g., detailed dietary cultural patterns, marital status, parity) was not systematically captured. Tenth, given the very large sample size of Cohort 1, additional weak correlations (|r| < 0.20) reached statistical significance but are unlikely to be of clinical relevance and have been flagged accordingly. These findings should be validated prospectively in multi-centre studies with functional assessment and complete biochemical data.

## 5. Conclusions

This study of 29,544 patients with obesity, complemented by integrative analysis of metabolic biomarkers (n = 2019), quantitative habitual dietary intake (*n* = 423) and directly measured inflammatory biomarkers, adipokines and serum 25(OH)–vitamin D (*n* = 116), demonstrates that integrative assessment of BIA-derived body composition and metabolic biomarkers provides a comprehensive framework for personalized prevention of obesity complications. The principal findings include the following: strong inverse associations between SMI and VFA (men: r = −0.832; women: r = −0.714; both *p* < 0.001, FDR q < 0.05) establish the muscle–fat axis as a central structural feature of obesity-related body composition. The body composition phenotype (low relative muscle mass + VFA ≥ 100 cm^2^) prevalence increased progressively with age (men: 24.6–42.0%; women: 10.3–31.8%), with the most pronounced differences observed at 50–59 years in men and 40–59 years in women. SMM–uric acid (r = +0.347), SMM–HDL-C (r = −0.321), and BMI–insulin (r = +0.233) associations (all *p* < 0.001, FDR q < 0.05) indicate that body composition parameters are associated with key metabolic biomarkers. Habitual dietary intake in the dietary subcohort showed a pattern of simultaneous saturated-fat excess (>70% of patients) and dietary-fibre deficit (>70% of patients), with sex-specific micronutrient gaps (vitamin B1 in both sexes: calcium and iron in women, with calcium intake declining significantly with age in women). Directly measured high-sensitivity CRP was elevated (median 5.59 mg/L) and significantly correlated with uric acid, triglycerides and HDL-C, providing direct evidence of chronic low-grade inflammation co-existing with the SMI–VFA structural axis. Based on observed cross-sectional patterns, preventive monitoring should begin at age 40 in both sexes, with intensification at age 50. These findings support body composition-based assessment of obesity, combined with quantitative dietary assessment and selective measurement of inflammatory biomarkers, as a complement to BMI-only evaluation and provide a framework for integrating BIA into routine clinical practice. Prospective interventional validation of the proposed framework is required.

## Figures and Tables

**Table 1:** Body composition parameters in the BIA cohort (*n* = 29,544).

Parameter	Total (*n* = 29,544)	Men (*n* = 8170)	Women (*n* = 21,374)	*p* (M vs. F)
Age, years	52.3 ± 12.0	51.8 ± 12.1	52.5 ± 11.9	0.012
Weight, kg	113.4 ± 24.1	127.5 ± 22.4	107.8 ± 22.3	<0.001
BMI, kg/m^2^	40.2 ± 7.9	39.8 ± 7.6	40.4 ± 8.0	0.038
Body fat, %	46.8 ± 6.9	39.2 ± 6.1	49.8 ± 4.8	<0.001
Fat mass, kg	54.1 ± 17.2	51.3 ± 17.5	55.2 ± 17.0	<0.001
SMM, kg	33.0 ± 7.7	41.8 ± 5.9	29.5 ± 4.6	<0.001
FFM, kg	60.7 ± 12.3	72.4 ± 9.8	56.1 ± 8.4	<0.001
VFA, cm^2^	228.9 ± 48.8	222.6 ± 50.1	231.4 ± 48.0	<0.001
ECW/TBW	0.384 ± 0.010	0.381 ± 0.009	0.386 ± 0.010	<0.001
BMR, kcal/day	1649 ± 275	1916 ± 227	1544 ± 180	<0.001
SMI, %	29.5 ± 4.6	33.4 ± 4.9	28.2 ± 2.8	<0.001

Data presented as M ± SD. Mann–Whitney U test for sex comparison. BMI, body mass index; SMM, skeletal muscle mass; FFM, fat-free mass; VFA, visceral fat area; ECW, extracellular water; TBW, total body water; BMR, basal metabolic rate; SMI, skeletal muscle index.

**Table 2:** Key BIA–biochemistry associations (*n* = 2019 paired observations).

BIA Parameter	Biomarker	r	*p*	FDR q
SMM	Uric acid	+0.347	<0.001	<0.05
SMM	HDL-C	−0.321	<0.001	<0.05
BMI	Insulin	+0.233	<0.001	<0.05
VFA	Insulin	+0.217	<0.001	<0.05
BMI	Glucose	+0.130	<0.001	<0.05
VFA	Glucose	+0.127	<0.001	<0.05
BMI	HDL-C	−0.151	<0.001	<0.05
SMM	Triglycerides	+0.158	<0.001	<0.05
BMI	Uric acid	+0.118	<0.001	<0.05
ECW/TBW	LDL-C	−0.143	<0.001	<0.05

Spearman correlations. Effect-size labels follow Cohen’s conventions (|r| < 0.20 weak; 0.20–0.40 weak–moderate; 0.40–0.60 moderate). Weak associations should be interpreted with caution despite reaching statistical significance, owing to the very large sample size. Abbreviations: BMI, body mass index; ECW, extracellular water; HDL-C, high-density lipoprotein cholesterol; LDL-C, low-density lipoprotein cholesterol; SMM, skeletal muscle mass; TBW, total body water; VFA, visceral fat area.

**Table 3:** Habitual nutrient intake in the dietary subcohort (*n* = 423): comparison of men and women.

Nutrient	n M	Men: Me [Q1; Q3]	n F	Women: Me [Q1; Q3]	*p*
Energy, kcal/day	100	2920.5 [2153.9; 3834.8]	323	2220.7 [1772.2; 3133.4]	<0.001
Protein, g/day	100	115.3 [80.4; 151.3]	322	94.3 [67.3; 126.4]	0.002
Fat, g/day	100	145.4 [105.6; 193.7]	322	114.0 [84.8; 160.9]	<0.001
Carbohydrate, g/day	99	241.8 [189.0; 370.2]	319	199.0 [138.6; 283.3]	<0.001
Saturated fat, g/day	92	49.5 [30.0; 66.8]	266	35.4 [25.9; 49.0]	<0.001
Omega-3, g/day	48	1.6 [0.7; 2.5]	135	1.4 [0.5; 2.8]	0.649
Cholesterol, mg/day	48	410.1 [227.6; 924.8]	135	349.0 [212.2; 651.9]	0.190
Dietary fibre, g/day	100	11.5 [7.9; 16.7]	322	10.6 [7.2; 16.6]	0.491
Calcium, mg/day	92	937.0 [668.3; 1446.3]	258	918.7 [635.7; 1308.7]	0.233
Magnesium, mg/day	48	306.1 [260.1; 441.4]	135	327.9 [239.5; 445.9]	0.890
Iron, mg/day	48	15.7 [13.5; 20.7]	135	15.2 [11.7; 20.1]	0.310
Vitamin B1, mg/day	65	1.3 [0.8; 2.0]	188	1.1 [0.7; 1.5]	0.010
Vitamin C, mg/day	21	185.4 [101.6; 284.1]	56	212.9 [149.7; 331.8]	0.478

Mann–Whitney U test. Habitual intake was assessed using the Russian Federal Research Centre software-based questionnaire “Scientific Nutrition Analysis Tool”. Reference daily intake values were from MP 2.3.1.0253-21.

**Table 4:** Proportion of patients with intake below 80% (deficiency) or above 120% (excess) of Russian recommended values (MP 2.3.1.0253-21), by sex.

Nutrient	Men: Deficit %	Men: Excess %	Women: Deficit %	Women: Excess %
Energy	21.0	53.0	16.7	44.0
Protein	17.0	68.0	12.4	67.4
Fat	10.0	77.0	4.3	75.2
Saturated fat	15.2	71.7	9.0	70.7
Omega-3	41.7	39.6	48.1	42.2
Cholesterol	27.1	60.4	30.4	48.9
Dietary fibre	85.0	2.0	72.7	9.6
Calcium	35.9	37.0	38.0	29.5
Magnesium	52.1	16.7	48.9	20.0
Iron	2.1	79.2	45.2	19.3
Vitamin B1	46.2	27.7	59.6	15.4
Vitamin B2	40.0	35.4	42.6	29.8
Niacin	40.0	33.8	45.6	22.8
Vitamin C	14.3	71.4	7.1	83.9

**Table 5:** Inflammatory biomarkers, adipokines, fasting insulin and serum 25(OH)-vitamin D in adults with obesity.

Biomarker	n	Median	Q1	Q3	Reference/Cut-Off
CRP, mg/L	59	5.59	2.31	8.59	<3 (low CV risk)
IL-6, pg/mL	31	6.61	4.03	9.69	<7
Leptin, ng/mL	32	53.65	43.08	75.85	Women 5–25; men 2–10
Adiponectin, μg/mL	33	14.30	9.90	19.00	6–20
Insulin, μIU/mL	82	16.10	12.20	20.62	2.6–25
Total IgE, IU/mL	73	62.50	22.00	150.00	<100
25(OH)D, ng/mL	36	25.15	19.20	31.38	≥30 (sufficiency)

**Table 6:** Hormonal status of patients with obesity, by sex and age group, Me [Q1; Q3].

Hormone	Sex	30–39 Years	40–49 Years	50–59 Years	*p*
Insulin, μIU/mL	M	10.7 [8.2; 15.6]	12.5 [7.8; 20.6]	11.7 [7.4; 18.5]	0.519
Insulin, μIU/mL	F	9.87 [6.62; 16.12]	9.30 [6.01; 13.55]	10.1 [6.4; 14.7]	0.214
TSH, μIU/mL	M	1.57 [1.08; 2.21]	1.46 [1.01; 2.06]	1.46 [0.97; 2.06]	0.538
TSH, μIU/mL	F	1.63 [1.18; 2.34]	1.62 [1.11; 2.32]	1.62 [1.08; 2.37]	0.883
Parathyroid hormone, pg/mL	M	42.2 [29.6; 58.5]	41.2 [32.0; 54.4]	45.3 [36.8; 59.9]	0.185
Parathyroid hormone, pg/mL	F	41.8 [29.4; 52.7]	41.1 [32.0; 52.8]	43.3 [34.0; 57.4]	0.038
Prolactin, mIU/L	M	214.5 [160.5; 279.5]	179.0 [141.8; 220.2]	185.0 [142.0; 255.5]	0.109
Prolactin, mIU/L	F	279.0 [207.0; 414.5]	268.0 [184.5; 375.5]	198.0 [146.2; 264.8]	<0.001
Oestradiol, pmol/L	F	55.0 [35.5; 113.9]	54.9 [29.8; 100.4]	16.8 [10.2; 24.7]	<0.001
Testosterone, nmol/L	M	9.71 [6.96; 12.99]	9.78 [6.92; 13.04]	9.96 [7.65; 13.68]	0.536
Testosterone, nmol/L	F	1.34 [1.06; 1.68]	1.60 [1.21; 2.70]	—	0.181

Kruskal–Wallis test across age groups within each sex.

**Table 7:** Micronutrient status of patients with obesity, by sex and age group, Me [Q1; Q3] (n = 2019).

Micronutrient	Sex	30–39 Years	40–49 Years	50–59 Years	*p*
25(OH)D, ng/mL	M	29.0 [22.8; 35.0]	29.8 [20.2; 35.4]	27.4 [15.6; 34.0]	0.377
25(OH)D, ng/mL	F	32.3 [24.1; 39.9]	29.3 [21.6; 38.6]	29.6 [21.9; 37.3]	0.196
Vitamin B12, pg/mL	M	279.0 [225.0; 510.0]	387.0 [295.8; 523.0]	378.0 [239.2; 554.8]	0.545
Vitamin B12, pg/mL	F	346.5 [229.5; 561.0]	306.0 [221.0; 447.5]	350.0 [267.0; 507.0]	0.123
Folate, ng/mL	M	6.71 [3.65; 9.63]	9.20 [6.18; 15.08]	7.42 [4.74; 12.60]	0.167
Folate, ng/mL	F	9.86 [5.71; 12.85]	9.59 [5.37; 13.89]	9.00 [6.08; 12.88]	0.962
Serum iron, μmol/L	M	19.9 [15.6; 24.5]	20.8 [16.0; 24.8]	21.4 [16.1; 26.7]	0.421
Serum iron, μmol/L	F	18.4 [12.6; 23.8]	18.5 [13.2; 23.8]	17.9 [13.7; 22.4]	0.821
Calcium, mmol/L	M	2.39 [2.28; 2.45]	2.46 [2.38; 2.54]	2.44 [2.40; 2.48]	0.061
Calcium, mmol/L	F	2.40 [2.35; 2.48]	2.37 [2.31; 2.44]	2.42 [2.34; 2.49]	<0.001
Magnesium, mmol/L	M	0.86 [0.81; 0.91]	0.85 [0.81; 0.89]	0.83 [0.79; 0.87]	0.523
Magnesium, mmol/L	F	0.85 [0.80; 0.88]	0.83 [0.79; 0.88]	0.85 [0.82; 0.89]	0.124

**Table 8:** Phenotype-specific nutritional recommendations for adults with obesity, derived from ESPEN/EASO guidelines and supported by observed dietary patterns in the present cohort.

Parameter	Men (≥40 Years)	Women (40–50 Years, Peri-Menopausal)
Protein	1.2–1.5 g/kg actual BW/day (1.6–2.0 g/kg ideal BW); leucine-rich sources (dairy, whey, eggs, soy, poultry)	1.0–1.2 g/kg actual BW/day (1.2–1.5 g/kg ideal BW); complete proteins (dairy, eggs, fish, soy); attention to vitamin B12
Carbohydrates	50–58% of energy; low-GI sources (whole grains, legumes, non-starchy vegetables); restrict added sugars	50–55% of energy; low-GI sources; fibre ≥25 g/day; restrict added sugars
Fats	25–30% of energy; omega-3 (EPA + DHA 500–1000 mg/day); saturated fat < 10% of energy	≈30% of energy; omega-3 (EPA + DHA 250–500 mg/day); emphasis on monounsaturated fats
Energy deficit	500 kcal/day below TEE (target 0.5–1.0 kg/week)	500 kcal/day below TEE (target 0.5–1.0 kg/week)
Hydration	1.8–2.3 L/day	1.5–2.0 L/day
Resistance exercise *	2–3 sessions/week	2–3 sessions/week
Special considerations	Uric acid: limit purine-rich foods, fructose, alcohol; hepatic support for MASLD prevention	Bone health: calcium 1000 mg/day; vitamin D 800 IU/day; soy isoflavones 50–100 mg/day; meal-timing optimisation

* Resistance-training and other physical-activity recommendations are adapted from ESPEN/EASO clinical guidelines [9,20]. Habitual physical activity in this cohort was uniformly very low (occupational category I, physical activity coefficient 1.4 × BMR); therefore, structured resistance-training recommendations are based on guidelines rather than on observed within-cohort variation.

**Table 9:** Three-Stage Age-Specific Prevention Framework for Metabolic Complications in Obesity (proposed).

Stage	Age/Trigger	Men	Women
Stage 1: Screening	From 30 yrs (men)/40 yrs (women)	Mandatory BIA + biochemistry + habitual-diet questionnaire at obesity diagnosis	Mandatory BIA + biochemistry + habitual-diet questionnaire at obesity diagnosis
Stage 2: Active Prevention	From 40 yrs (both sexes)	Adequate protein (1.2–1.5 g/kg/day); omega-3 (500–1000 mg/day); low-GI carbohydrates; hydration ≥2 L/day; purine restriction; resistance training 2–3×/wk *; annual BIA + biochemistry + diet review	Low-GI carbohydrates; Mediterranean-pattern diet; protein 1.0–1.2 g/kg/day; calcium 1000 mg/day; vitamin D 800 IU/day; resistance training 2–3×/wk *; annual BIA + biochemistry + diet review
Stage 3: Intensified Prevention	From 50 yrs (both sexes)	All Stage 2 measures + semi-annual BIA; consider higher protein (1.5 g/kg/day); intensified hepatic and uric-acid monitoring; functional muscle testing	All Stage 2 measures + semi-annual BIA; soy isoflavones 50–100 mg/day; vitamin B12 monitoring; meal-timing optimisation; functional muscle testing

* Resistance-training recommendations are adapted from ESPEN/EASO guidelines [9,20].

## Data Availability

The data presented in this study are available upon reasonable request from the corresponding author. The data are not publicly available due to privacy and ethical restrictions.

## Abbreviations

BIA	Bioelectrical Impedance Analysis
BMI	Body Mass Index
ECW	Extracellular Water
FFM	Fat-Free Mass
FNIH	Foundation for the National Institutes of Health
FDR	False Discovery Rate
HDL-C	High-Density Lipoprotein Cholesterol
HbA1c	Glycated Haemoglobin
HOMA-IR	Homeostatic Model Assessment for Insulin Resistance
LDL-C	Low-Density Lipoprotein Cholesterol
MASLD	Metabolic Dysfunction-Associated Steatotic Liver Disease
SMI	Skeletal Muscle Index
SMM	Skeletal Muscle Mass
TBW	Total Body Water
VFA	Visceral Fat Area
